# Application of the COM-B model to barriers and facilitators to chlamydia testing in general practice for young people and primary care practitioners: a systematic review

**DOI:** 10.1186/s13012-018-0821-y

**Published:** 2018-10-22

**Authors:** Lorraine K McDonagh, John M Saunders, Jackie Cassell, Tyrone Curtis, Hamad Bastaki, Thomas Hartney, Greta Rait

**Affiliations:** 1 Research Department of Primary Care and Population Health, University College London, London, NW3 2PF UK; 2 National Institute for Health Research, Health Protection Research Unit in Blood Borne and Sexually Transmitted Infections, University College London, London, NW3 2PF UK; 3 National Chlamydia Screening Programme, Public Health England, London, NW9 5EQ UK; 4 Department of Primary Care and Public Health, Brighton and Sussex Medical School, University of Brighton, Brighton, BN1 9PH UK; 5 Centre for Population Research in Sexual Health and HIV, Institute for Global Health, University College London, London, WC1E 6JB UK

**Keywords:** Implementation, Chlamydia, General practice, Primary care, Young people, Systematic review

## Abstract

**Background:**

Chlamydia is a major public health concern, with high economic and social costs. In 2016, there were over 200,000 chlamydia diagnoses made in England. The highest prevalence rates are found among young people. Although annual testing for sexually active young people is recommended, many do not receive testing. General practice is one ideal setting for testing, yet attempts to increase testing in this setting have been disappointing. The Capability, Opportunity, and Motivation Model of Behaviour (COM-B model) may help improve understanding of the underpinnings of chlamydia testing. The aim of this systematic review was to (1) identify barriers and facilitators to chlamydia testing for young people and primary care practitioners in general practice and (2) map facilitators and barriers onto the COM-B model.

**Methods:**

Qualitative, quantitative, and mixed methods studies published after 2000 were included. Seven databases were searched to identify peer-reviewed publications which examined barriers and facilitators to chlamydia testing in general practice. The quality of included studies was assessed using the Critical Appraisal Skills Programme. Data (i.e., participant quotations, theme descriptions, and survey results) regarding study design and key findings were extracted. The data was first analysed using thematic analysis, following this, the resultant factors were mapped onto the COM-B model components. All findings are reported in accordance with the Preferred Reporting Items for Systematic Reviews and Meta-Analyses (PRISMA) guidelines.

**Results:**

Four hundred eleven papers were identified; 39 met the inclusion criteria. Barriers and facilitators were identified at the patient (e.g., knowledge), provider (e.g., time constraints), and service level (e.g., practice nurses). Factors were categorised into the subcomponents of the model: physical capability (e.g., practice nurse involvement), psychological capability (e.g.: lack of knowledge), reflective motivation (e.g., beliefs regarding perceived risk), automatic motivation (e.g., embarrassment and shame), physical opportunity (e.g., time constraints), social opportunity (e.g., stigma).

**Conclusions:**

This systematic review provides a synthesis of the literature which acknowledges factors across multiple levels and components. The COM-B model provided the framework for understanding the complexity of chlamydia testing behaviour. While we cannot at this juncture state which component represents the most salient influence on chlamydia testing, across all three levels, multiple barriers and facilitators were identified relating psychological capability and physical and social opportunity. Implementation should focus on (1) normalisation, (2) communication, (3) infection-specific information, and (4) mode of testing. In order to increase chlamydia testing in general practice, a multifaceted theory- and evidence-based approach is needed.

**Trial registration:**

PROSPERO CRD42016041786

**Electronic supplementary material:**

The online version of this article (10.1186/s13012-018-0821-y) contains supplementary material, which is available to authorized users.

## Introduction

*Chlamydia trachomatis* (chlamydia) is the most commonly diagnosed bacterial sexually transmitted infection (STI) in England with 202,546 diagnoses in 2016, 63% of which were among 15 to 24-year-olds [1]. Chlamydia is often asymptomatic; therefore, testing and treatment are essential to prevent transmission and potential negative reproductive health outcomes [2]. Chlamydia can be tested for using a genital or vulvo-vaginal swab (self-administered or health care professional-administered) or a urine sample. Laboratory diagnosis is conducted using nucleic acid amplification tests (NAATs) which allow the use of non-invasive samples (i.e., urine and self-taken vulvo-vaginal swabs). General practice presents an ideal setting for testing. Over 60% of young people visit general practice annually [3, 4] and report a preference to receive testing and results from a general practitioner (GP) [5–8]. Positivity is higher in general practice than non-healthcare settings such as universities [9, 10], while regular screening is facilitated by attendance for other reasons [9].

England’s National Chlamydia Screening Programme (NCSP) advocates opportunistic testing (i.e., testing regardless of reason for attendance such as those who present with other ailments such as the common cold) across a range of settings (including general practice). The NCSP also recommends testing for sexually active young people annually and on change of sex partner. In 2016, approximately 25% of 15–24-year-olds in England were tested for chlamydia but only 19% of tests were conducted in general practice [11]. A narrative review reported commonly cited barriers to testing in general practice (from the perspectives of both primary care practitioners [PCP] and young people) which include stigma, poor knowledge/training, and time constraints [12]. This review, however, was conducted in 2013, and several new studies in this area have been published since. Furthermore, facilitators to testing are not well investigated, and interventions to increase testing in general practice have been disappointing [13–17]. One conceivable explanation for these disappointing results is the lack of input from theories of behaviour.

**Fig. 1:**
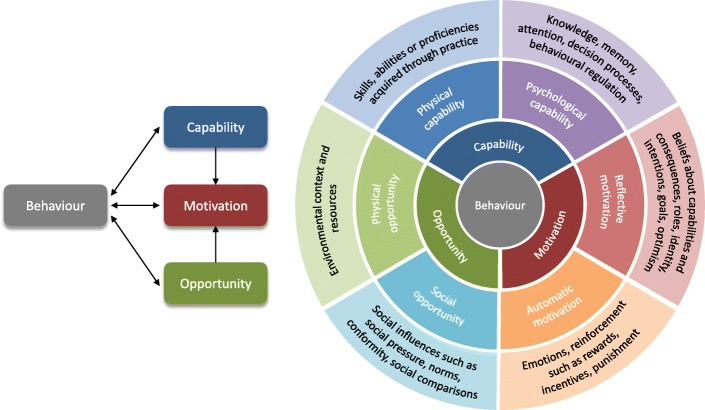
The COM-B Model [15]

Implementing changes in general practice requires behaviour change in several agents (e.g., PCP, patients, commissioners) [18] underpinned by a theoretical understanding of the behaviour [19, 20]. The Capability, Opportunity, Motivation, Behaviour (COM-B) model is one theory of behaviour which can contribute insights into chlamydia testing behaviour [20]. COM-B posits behaviour as the result of an *interaction* between three components: capability, opportunity, and motivation (see Fig. 1). Capability can be psychological (knowledge) or physical (skills); opportunity can be social (societal influences) or physical (environmental resources); motivation can be automatic (emotion) or reflective (beliefs, intentions).

The benefit of employing the COM-B Model over a single theory of behaviour is that several distinct explanatory components are outlined; thus, additional potential influences on behaviour can be considered. COM-B lies at the centre of the Behaviour Change Wheel (BCW), a tool kit for designing behaviour change interventions [20]), and is the starting point of intervention development. COM-B components can be mapped onto the BCW and the Behaviour Change Technique Taxonomy which facilitates the selection of intervention strategies that are likely to be appropriate and effective in addressing the barriers and facilitators for each component. This model has been effectively applied to many health behaviours at both individual and organisational levels [21–28], but not yet to chlamydia testing.

It remains unclear how to meaningfully translate our understanding of barriers and facilitators to testing into clinical practice. While the COM-B model has primarily been applied to intervention design, its associated Theoretical Domains Framework (division of COM-B components into 14 theoretical domains [18]) has recently been applied as a synthesis framework for systematic reviews in other contexts [29–31]. Hence, the COM-B model could also provide a helpful framework for evidence synthesis in a systematic review.

The application of COM-B to factors associated with the implementation of chlamydia testing in general practice will enable us to develop a coherent framework for understanding chlamydia testing, focussed on identifying appropriate behaviour change techniques to improve implementation and increase chlamydia testing [32]. The aim of this systematic review was to (1) identify barriers and facilitators to chlamydia testing for young people in general practice and (2) map these onto the COM-B model.

## Methods

The protocol, published elsewhere [33], is summarised briefly here. This review was conducted according to PRISMA guidelines [34] (see Additional file 1) and registered with the International Prospective Register of Systematic Reviews (CRD42016041786).

### Eligibility criteria

Eligible studies had to explore facilitators and/or barriers to chlamydia testing, views towards testing, and/or acceptability of testing in general practice. A barrier was defined as a factor that obstructs or prevents chlamydia testing; a facilitator was defined as a factor that supports or promotes testing. Table 1 summarises the inclusion and exclusion criteria.

**Table 1:** List of inclusion/exclusion criteria

Inclusion criteria	Exclusion criteria
Population: young people (aged 15–24 years) and primary care providers (PCP; general practitioners, practice nurses, nurse practitioners)	Population: exclusively on commercial sex workers, incarcerated people, people living with HIV, victims of sexual or domestic abuse or violence, intravenous drug users, and individuals with no fixed address
Randomised and non-randomised controlled trials, pre- and post-test designs, non-experiment observational (cross-sectional, case-series, case studies), qualitative (interviews, focus groups), and mixed method paper	Commentary or opinion publications that did not present new data
Conducted in countries where the model of delivering healthcare in general practice is comparable to the UK (Australia, Denmark, Ireland, Netherlands, and New Zealand) where (1) the GP acts as a gatekeeper to access specialist services and (2) general practice services are publicly financed	Conducted in countries where the healthcare system and general practice setting is not comparable to that of the UK (i.e., USA, Canada) because (1) the role of the GP in these countries differs and specialist services are readily accessible without initial GP contact and (2) most healthcare is delivered privately meaning many have to pay out-of-pocket for insurance and care. Consequently, these different systems will have distinct characteristics and influential barriers and facilitators beyond the scope of this review
Opportunistic and systematic testing in general practice	Exclusively set outside of general practice, exclusively focused on partner notification, campaigns exclusively focused on health promotion, and testing for diagnostic purposes when symptoms are present

### Search strategy

**Fig. 2:**
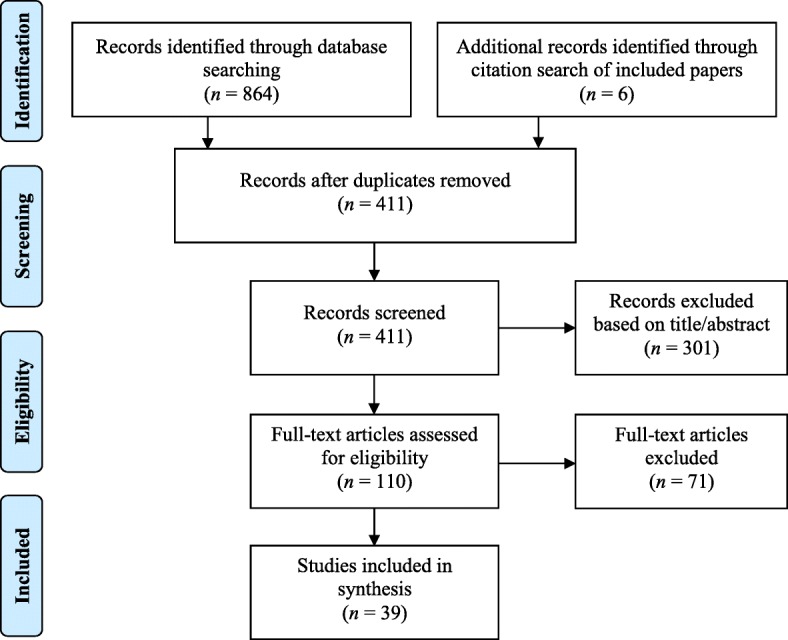
Flowchart illustrating the process of inclusion and exclusion of papers in the study

Seven databases (MEDLINE, PubMed, Embase, Informit, Web of Science, PsycINFO, Scopus) were searched from January 2000 to April 2018. Pre-2000 studies excluded as NAATs were introduced around this time, thus widening testing to non-clinical settings. The search strategy is presented Additional file 2. Three sets of search terms were developed relating to the context (general practice), intervention (chlamydia testing), and outcomes (barriers, facilitators) [33]. Figure 2 illustrates the selection process.

### Data extraction

A standardised framework was devised and used to record the aims, methodological characteristics (e.g., design, data collection, participants), theoretical framework employed (if any), main findings (i.e., participant quotations, themes identified by article authors, and survey results), and conclusion of each study. Data extraction was undertaken by one reviewer (LMD) and checked by a second reviewer (HB/TH).

### Quality assessment

The quality of each paper was independently assessed using the Critical Appraisal Skills Programme (CASP [35]) by two reviewers (LMD and HB/TH). As per recommendations for use, the tools were not used to score individual studies as such but used as a broad guide to provide a context in which to interpret findings. Because the aim was to describe and synthesise the literature, this process was not used to exclude papers.

### Data synthesis and analysis

Study characteristics and outcomes were summarised in an evidence table. First, thematic analysis [36] was used to identify prominent themes. Themes were refined through discussion and the use of constant comparison within and between codes to ensure that they accurately reflected the material.

Second, the identified themes were classified into the six sub-components of the COM-B model described above (Fig. 1). Data classification, following expert guidelines [20], was conducted by one reviewer (LMD) in consultation with members of the review team at multiple data-synthesis meetings (JS, JC, GR). Discrepancies were resolved by consensus.

## Results

**Fig. 3:**
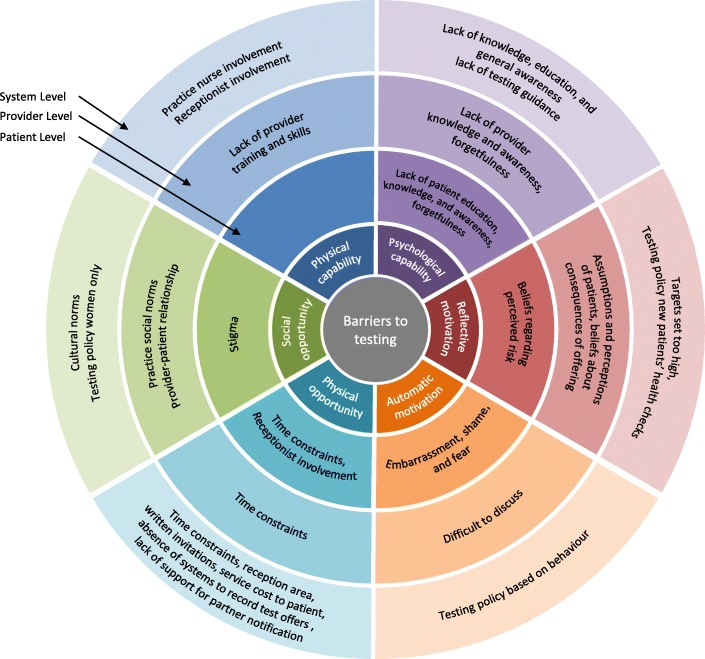
Barriers to chlamydia testing at system, provider, and patient levels mapped on to the subcomponents of COM-B model

**Fig. 4:**
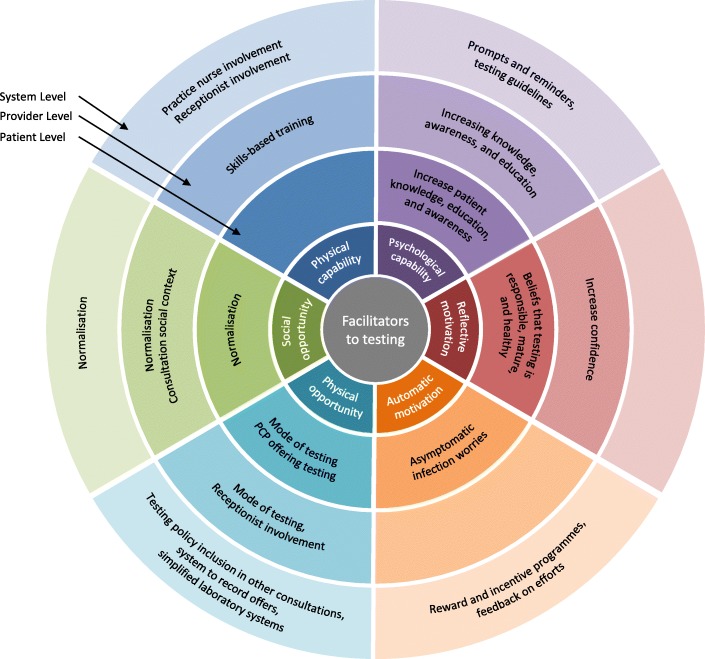
Facilitators to chlamydia testing at system, provider, and patient levels mapped on to the subcomponents of COM-B model

Thirty-nine papers met the inclusion criteria; 14 focused on patients [37–50] (Table 2) and 25 on providers [51–75] (Table 3). Barriers (Fig. 3) and facilitators (Fig. 4) were identified at patient, provider, and service level (i.e., factors stemming from the broader healthcare system), with some factors spanning all three levels. Most studies did not use any theory, only seven studies used any behavioural theory; six used the Theory of Planned Behaviour [41, 48, 50, 66, 73, 75] and one used Normalisation Process Theory [70]. Some studies were qualitative evaluations of trials that had used theory for intervention development [48, 70, 75], others used theory to guide interview questions and questionnaires [41, 66, 73] or provide a framework for results [50, 70]. When judged against the CASP criteria, the majority of studies were methodologically sound, except three [44, 46, 71] which lacked detail on several areas (e.g., recruitment strategies, rigour of data analysis). The full quality assessment is available upon request. Table 4 provides an overview of all results. The detailed findings of included studies are provided in Additional files 3 (providers) and 4 (young people), with illustrative quotes for each theme presented in Additional file 5.

**Table 2:** Characteristics of included studies with primary care professionals (PCP)

Author	Location	Participants	Design	Theory	Method	Analysis
Allison et al. [74]	UK (England)	26 general practice staff (9 GPs; 13 PNs; 3 practice managers; 1 receptionist) who had participated in an intervention (m = 5; f = 23)	Qualitative	None	Semi-structured interviews	Modified framework analysis
Bilardi et al. [51]	Australia	43 GPs; intervention group *n* = 20 (m = 9; f = 11); control group *n* = 23 (m = 11; f = 12); age range = < 35–55+ years	Quantitative	None	Questionnaire following pilot cluster RCT	Descriptive (percentages) and mixed-effects logistic regression
Bilardi et al. [52]	Australia	14 GPs (m = 6; f = 8) Age: 31–40 years = 4; 41–50 years = 4; 51–60 years = 6	Quantitative	None	Questionnaire following pilot RCT; interviewer-administered, open ended	Test for equality in proportion and thematic analysis
Calamai et al. [53]	UK	55 GPs and PNs (m = 13; f = 42)	Quantitative	None	Questionnaire	Descriptives: frequencies
Freeman et al. [54]	UK (England)	156 healthcare staff from 25 practices (72 GPs; 46 PNs; 8 practice managers; 23 administrators and receptionists; others)	Qualitative	None	Focus groups	Stepwise framework analytical approach (inductive)
Hocking et al. [55]	Australia	GPs (*n* = 21 interview; *n* = 225 questionnaires); mean age = 49.8 years	Mixed: qualitative and quantitative	None	Semi-structured interviews and postal questionnaire	Thematic analysis and descriptive statistics
Khan et al. [56]	Australia	409 GPs (m = 233; f = 176)	Quantitative	None	Questionnaire (paper, postal)	Correlation analysis, logistic regression
Lorch et al. [60]	Australia	556 GPs (m = 338; f = 218) and 118 PNs (m = 2; f = 116) from 143 clinics; age range = 30–59 years	Quantitative	None	Questionnaire (paper)	Descriptives, regression
Lorch et al. [57]	Australia	72 PNs (m = 1; f = 71)	Quantitative	None	Questionnaire	Chi-squared paired *t* test
Lorch et al. [58]	Australia	44 GPs (m = 27; f = 16)	Qualitative	None	Semi-structured interviews	Thematically using content analysis
Lorch et al. [59]	Australia	23 PNs (m = 1; f = 22); age range = 30–59 years	Qualitative	None	Semi-structured interviews	Thematically using content analysis
Lorimer et al. [61]	UK (Scotland)	18 GPs and 8 PNs	Qualitative	None	Semi-structured interviews (telephone)	Framework analysis with thematic coding
Ma and Clarke [62]	UK (England)	4 consultants in sexual and reproductive health, 1 consultant in public health, 1 chlamydia screening coordinator, 3 GPs and 3 PNs	Qualitative	None	Semi-structured interviews	Variation of thematic analysis
McKernon and Azariah [63]	New Zealand	76 staff participating in pilot trial: 5 receptionists, 5 clinical assistants, 24 nurses, 31 doctors, 10 practice managers (who were also doctors), and 4 operations managers	Quantitative	None	Questionnaire	Descriptives
McNulty et al. [64]	UK (England)	12 focus groups of GPs, PNs, practice managers, midwives, and district nurses (total *n* not reported)	Qualitative	None	Focus groups	Modified grounded theory approach utilising the constant comparative method
McNulty et al. [65]	UK (England)	General practice staff (GPs, PNs) from high/low testing rates and rural/urban areas (total *n* not reported)	Qualitative	None	Focus groups	Thematic analysis using constant comparative method
McNulty et al. [66]	UK (England)	Focus groups: 72 GPs, 46 PNs, 23 receptionists and administrators, 8 practice managers, 7 other staff. Interviews: 5 GPs, 3 nurses, 1 receptionist, 2health care assistants, 1 manager.	Qualitative	Theory of Planned Behaviour	Semi-structured interviews (12) and focus groups (25)	Stepwise framework analytical approach
McNulty et al. [67]	UK (England)	9 chlamydia screening co-ordinators from areas with significant screening in general practice	Qualitative	None	Semi-structured interviews (telephone)	Interpretative phenomenological thematic approach
McNulty et al. [75]	UK (England), Estonia, Sweden, France	45general practice staff, 18 stakeholders, 13 trainers (England 25, Estonia 15, France 23; Sweden 13)	Qualitative	Theory of Planned Behaviour	Semi-structured interviews	Thematic analysis
Merritt et al. [68]	Australia	10 GPs from 6 practices	Uncontrolled before and after trial	None	Meetings every 2 month during intervention	Descriptive statistics
Perkins et al. [69]	UK (England)	13 GPs; 14 PNs; 15 practice receptionists; 11 practice managers	Qualitative	None	Semi-structured interviews	Open-coding method
Ricketts et al. [70]	UK (England)	29 general practice staff: 9 GPs; 13 PNs; 7 receptionists; from 8 high and low 7 screening intervention practices	Qualitative (evaluation of intervention)	Normalisation Process Theory	Semi-structured interviews	Thematic analysis (within a Normalisation Process Theory Framework)
Robertson and Williams [71]	UK (Wales)	PNs (7 qualitative; 33 quantitative)	Mixed: qualitative and quantitative	None	Semi-structured interviews and questionnaire	Descriptive statistics
Senok et al. [72]	UK (Scotland)	13 GP’s, PNs and administrative staff	Feasibility study for a RCT and qualitative	None	In-depth interviews	Thematic analysis
Wallace et al. [73]	UK (England)	General practice staff 12 interviews; 5 GPs; 3 PNs; 1 practice manager; 3 receptionists. 55 questionnaires (m = 5; f = 50); 18 GPS; 26 PNs; 9 receptionists; 1 practice manager; 1 research nurse	Mixed: qualitative and quantitative	Theory of Planned Behaviour	Questionnaire (paper = 52; online = 3)	Quantitative: frequencies, *t* tests, chi-square tests Qualitative: thematic analysis

*f* female, *GP* general practitioner, *m* male, *PN* practice nurse, *RCT* randomised controlled trial

**Table 3:** Characteristics of included studies with young people (YP)

Author	Location	Participants	Design	Theory	Method	Analysis
Balfe et al. [37]	Ireland	30 YP attending health services for STI test (m = 9 [MSM = 3]); f = 21); age range = 18–29	Qualitative	None	Semi-structured interviews	Thematic analysis
Balfe et al. [38]	Ireland	35 young women; late teens to late 20s	Qualitative	None	Semi-structured interviews	Not reported
Brugha et al. [39]	Ireland	6085 YP attending 5 community healthcare settings and 1 GUM clinic, over a 2-week period (m = 2379; f = 3706); age range = 18–29	Quantitative	None	Questionnaire	Descriptive statistics: frequencies and × 2 cross-tabulations with two-tailed tests
Ewert et al. [49]	Australia	28 young men who were university students, age range = 18–25 (mean age = 20.8)	Qualitative	None	Semi-structured interviews	Content and thematic analysis
Heritage and Jones [40]	UK (England)	18 YP; 12 via schools, 6 via GP practice (m = 6; f = 12); age range = 16–18	Qualitative	None	Semi-structured interviews (2) and focus groups (*n* = 16)	Long-table approach (quotes categorised according to questions)
Hogan et al. [41]	Ireland	36 YP attending general practice (m = 9; f = 27); age range = 15–24 (mean age = 21)	Qualitative	Theory of Planned Behaviour	Semi-structured interviews	Thematic analysis
Jones et al. [48]	UK (England)	30 young people (m = 9; f = 21) attending general practice; age range 16–24	Qualitative	Theory of Planned Behaviour	Semi-structured interviews	Thematic framework
Mac Phail et al. [47]	New Zealand	956 university students (m = 272; f = 682, tg = 2); age range = 18–29	Quantitative	None	Questionnaire	Descriptive statistics
Mills et al. [42]	UK (England)	45 people registered with 27 general practices who returned postal test kits (m = 19; f = 26; positive = 25, negative = 20); age range = 16–39	Qualitative	None	Semi-structured interviews	Thematic analysis
Normansell et al. [50]	UK (England)	17 multi-ethnic women in further education college; age range = 16–25	Qualitative	Multiple: Theory of Planned Behaviour, Candidacy, Stigma	Semi-structured interviews	Thematic framework
Pavlin et al. [43]	Australia	24 young women; age range = 16–25	Qualitative	None	Semi-structured interviews	Thematic analysis
Pimenta et al. [44]	UK (England)	25 sexually active women attending healthcare settings for any reason (m = 1; f = 24); age range = 16–24	Qualitative	None	Semi-structured interviews	Content analysis
Santer et al. [45]	UK (Scotland)	Women: age ≤ 20 attending for contraception/pregnancy testing; ≤ 35 attending for cervical screening (positive = 4, negative = 14, awaiting = 2); age range = 15–31	Qualitative	None	Semi-structured interviews	Framework approach
Zakher and Kang [46]	Australia	185 university students (m = 40; f = 145); age range = 16–25 (mean age = 21)	Quantitative	None	Questionnaire	*t* tests, ANOVA, chi-square tests

*ANOVA* analysis of variance, *f* female, *GUM* genitourinary medicine, *m* male, *tg* transgender, *YP* young people

**Table 4:** Overview of results: Summary of barriers and facilitators across levels (patient, provider, and service) and theoretical component

COM-B Subcomponent	Patient Level				Provider Level				Service Level			
	Barrier	Source	Facilitator	Source	Barrier	Source	Facilitator	Source	Barrier	Source	Facilitator	Source
Physical capability					Lack of training and skills	[55, 57, 60, 63, 64, 66, 69, 73]	Skills-based training	[56, 59, 60, 62, 64, 65, 67, 70, 73–75]	Receptionist involvement	[38, 40, 41, 44, 48, 66, 67, 69, 70, 74, 75]	Receptionist involvement	[38, 40, 41, 44, 48, 66, 67, 69, 70, 74, 75]
									Practice nurse involvement	[58–60, 71]	Practice nurse involvement	[55, 58, 59, 63, 66, 69, 71]
Psychological capability	Lack of patient education, knowledge, and awareness	[51, 55, 60, 73]	Increase knowledge, education, and awareness	[37, 41, 43, 48, 49, 51, 54, 55, 61, 62, 64, 67, 68, 73]	Lack of provider knowledge and awareness	[55, 60, 63–66, 71, 75]	Increasing knowledge, awareness, and education	[54–56, 59, 62, 64, 65, 67, 70, 73, 75]	Lack of testing guidance	[64]	Prompts and reminders	[55, 66, 67, 70, 75]
	Forgetfulness	[41, 48, 75]			Forgetfulness	[51, 52, 55, 60, 66, 68, 70, 75]			Lack of knowledge, education, and general awareness	[55, 60, 63–66, 71]	Testing guidelines	[55, 60]
Reflective motivation	Beliefs regarding perceived risk	[37, 41, 42, 44–46]	Beliefs that testing is responsible, mature, and healthy	[37, 38]	Assumptions and perceptions of patients	[53–55, 61, 66, 69, 70, 74, 75]	Increase confidence	[59, 62, 70]	Targets set too high	[70, 74]		
					Beliefs about consequences of offering	[54, 66, 69, 70, 73, 74]			Testing policy: new patients’ health checks	[64]		
Automatic motivation	Embarrassment and shame	[37, 40–43, 48, 49, 55, 69, 70, 73] and [37, 38, 40, 42, 43]	Asymptomatic infection worries	[37, 44, 45]	Difficult to discuss	[52, 60–62, 64, 66, 68–70, 73, 75]			Testing policy: based on behaviour	[41, 48]	Reward and incentive programmes	[51, 55, 61, 62, 66–69, 74, 75]
	Fear	[38, 41–43, 48, 50]										
											Feedback on efforts	[70, 74, 75]
Physical opportunity	Time constraints	[40, 41, 48]	PCP offering testing	[41, 47]	Time constraints	[51, 52, 55, 57–64, 66, 68–75]	Mode of testing	[55, 62, 64, 73, 75]	Time constraints	[40, 41, 48] and [51, 52, 55, 57–64, 66, 68–75]	Promotional materials	[54, 63, 70, 74, 75]
	Receptionist involvement	[38, 40, 41, 44, 48, 66, 67, 69, 70, 74, 75]	Mode of testing	[39–41, 44, 48]			Receptionist involvement	[38, 40, 41, 44, 48, 66, 67, 69, 70, 74, 75]	Reception area	[38, 40, 41, 44, 48, 70] and [74, 75]	Testing policy: inclusion in other consultations	[48, 66, 67]
									Written invitations	[61, 67]	System to record offers	[66]
									Service cost to patient	[37, 39, 47, 60]	Simplified laboratory systems	[59, 62, 66, 68]
									Absence of systems to record test offers	[66, 69]	Support for partner notification	[62, 64, 69]
									Lack of support for partner notification	[55, 60, 62–64, 73]		
Social opportunity	Stigma	[37, 38, 40–43, 55, 61, 71]	Normalisation	[38, 41–43, 61]	Practice social norms	[66, 73]	Normalisation	[54, 61, 66, 67, 70, 74]	Testing policy: women only	[37, 38, 69]	Normalisation	[52, 54, 55, 61, 63, 66, 67, 70]
					Provider-patient relationship	[48, 55, 58, 59, 66, 74]	Consultation social context	[45, 55, 56, 61, 64, 67, 70, 73]	Cultural norms	[66, 73]	Testing policy: blanket testing	[38, 48]

*COM-B* capability, opportunity, motivation, behaviour

### Patient level factors

#### Barriers

##### Psychological capability

ᅟ

*Lack of patient (and public) education, knowledge, and awareness* [51, 55, 60, 73]. This was reported as a barrier by PCPs. One study of young people [41] reported lack of knowledge about chlamydia, its sequelae, and the screening process as barriers.

*Forgetfulness* [41, 48, 75]. While young people expressed enthusiasm for self-sampling, forgetting to return samples was reported as a barrier.

##### Reflective motivation

ᅟ

*Beliefs regarding perceived risk* [37, 41, 42, 44–46]. Young people who perceived themselves to be at low or no risk were less likely to test. These beliefs originated from perceived low chlamydia prevalence [45], the asymptomatic nature of chlamydia [37, 45], personal sexual history, and the perceived sexual history of a partner. Despite feeling positive about testing [37], there was no sense of urgency, so it was easy to avoid or postpone testing.

##### Automatic motivation

ᅟ

*Embarrassment* [37, 40–43, 48, 49, 55, 69, 70, 73] and *shame* [37, 38, 40, 42, 43]. The terms embarrassment and shame were used interchangeably. We considered these as distinct constructs (i.e., shame is an emotional response to something considered morally wrong whereas embarrassment does not imply wrongdoing [76, 77]) and tried to distinguish between these where possible. Embarrassment was cited by young people to explain their aversion to reception staff offering testing kits [40, 41] or having to walk through reception with a sample [48], being offered a test in front of parents [40, 48, 49], and returning self-sampling kits in case they were seen by someone they knew [41, 48, 49]. Embarrassment at having to undress in front of a PCP was also highlighted, particularly for young women [37]. Shame was associated with a positive chlamydia diagnosis [42, 43]. Men in one study expressed little embarrassment or shame at receiving a chlamydia diagnosis and perceived it as more of an issue for women [42]. Shame and embarrassment were experienced in relation to unprotected sex and a concern that a PCP would judge this behaviour [37, 41]. For some young women, the need to maintain a certain identity (e.g., “good girl” as opposed to a “bad girl”) was a barrier to testing [37, 38, 50] which was threatened by fear of being viewed as promiscuous or engaging in risky behaviour by a PCP [37]. Participants in one study suggested that having to provide a sexual history, particularly the number of sexual partners, was a barrier to testing [43].

*Fear* [38, 41–43, 48, 50]. Fear related to receiving a positive result [43, 50], having to tell previous partners [42], parents finding out [41, 48], and being judged by others [38, 41, 42, 50]. Some participants suggested that being afraid of receiving a positive result might deter people from testing in the first place [41].

##### Social opportunity

ᅟ

*Stigma* [37, 38, 40–43, 55, 61, 71]. The stigma of having an STI could outweigh the benefits of engaging in a healthy activity such as testing [38]. Some young women had preconceived negative views of the type of woman who would test for or be infected with chlamydia. Some participants were concerned that a chlamydia diagnosis would make others see them as sexually promiscuous and “dirty” [37, 38, 42, 43]. Being observed to have STI testing was similarly stigmatising [37, 38]. Participants were reluctant to accept a test in public locations (e.g., reception area) or to return self-sampling kits there, in case they were seen by someone they knew [39–41, 48].

#### Facilitators

##### Psychological capability

ᅟ

*Increasing knowledge, education, and awareness* [37, 41, 43, 48, 49, 51, 54, 55, 61, 62, 64, 67, 68, 73]. Information on chlamydia transmission, the testing process, risks of untreated infection, and ease of treatment were enablers of testing. The fear of receiving a positive result (automatic motivation barrier) could be outweighed by information on ease of treatment [43, 45]. Increasing awareness could be achieved through PCP discussion with patients [41], sexual health education in schools [49], public awareness campaigns [43, 49, 51, 55, 61, 62, 64, 67, 68], and promotional materials such as leaflets and posters [41, 54, 73]. PCPs also believed that increasing patient awareness enable testing, thereby reducing provider physical capability barriers [67, 73].

##### Reflective motivation

ᅟ

*Beliefs that testing is responsible, mature, and healthy* [37, 38]. Moral aspects of testing were raised, some saw testing as a moral practice and viewing it as the “right” or “good” (links to social opportunity), “mature” thing to do, and a responsible practice to engage in. Participants anticipated feeling guilty if they transmitted an infection so testing allowed the respondents to feel that they were protecting their own and partners’ health and bodies [37, 38].

##### Automatic motivation

ᅟ

*Asymptomatic infection worries* [37, 44, 45]. Some young people expressed concern about the damage an asymptomatic infection could have for their reproductive health which reportedly arose from contact with health promotion materials and individuals who had attended for chlamydia testing. As indicated above under the “Psychological capability” section, informing young people of the risks of asymptomatic infection can thus facilitate chlamydia testing.

##### Physical opportunity

ᅟ

*PCP offering testing* [41, 47]. Young people anticipated feeling uncomfortable asking for a test and would prefer it to be offered by their PCP.

*Mode of testing* [39–41, 44, 48]. Young people viewed self-sampling kits positively by young people as they allowed the test to be done in a more convenient and comfortable location (i.e., at home) [39] with urine samples preferred to vulval-vaginal swabs [40, 44]. The need for a discreet location and unsuitability of the reception was again highlighted (social opportunity barrier) [41, 48]. To minimise forgetting to return samples (psychological capability barrier), patients should complete samples prior to leaving the practice [75]—and many reported a preference for doing so [41, 48]. Self-sampling kits were also viewed positively by staff to reduce workload, time constraints (physical capability barrier), and for ease of use (e.g., [55, 62, 64]).

### Provider level factors

#### Barriers

##### Physical capability

ᅟ

*Lack of training and skills* [55, 57, 60, 63, 64, 66, 69, 73]. PCPs reported a lack of appropriate training and skills needed to discuss sexual health [64], take sexual history [55], offer a test [63], respond to a positive test and manage treatment [55], and conduct partner notification [60, 69]. This led to reduced confidence to offer testing (reflective motivation) and discuss sexual health with patients [55, 66]. In one study, young people felt that GPs lack sexual health expertise and thus preferred to attend sexual health clinics [50].

##### Psychological capability

ᅟ

*Lack of provider knowledge and awareness* [55, 60, 63–66, 71, 75]. Lack of knowledge about the epidemiology and presentation of chlamydia [55, 65], benefits of testing [64], at-risk populations such as young people [60], how to take specimens [64, 71, 75], and treatment options [63] were described. Practitioners who were unaware of the public health importance of testing and screening programmes would be less likely to find chlamydia testing a priority [66].

*Forgetfulness* [51, 52, 55, 60, 66, 68, 70, 75]. In some studies, PCPs only remembered to test when patients attended for other related health issues (e.g., contraception) or revealed high-risk behaviours [52]. Other PCPs remembered at the start of a trial or screening programme but forgot over time [51] and lack of a formal recall/reminder system to help staff remember was a barrier [55].

##### Reflective motivation

ᅟ

*Assumptions and perceptions of patients* [53–55, 61, 66, 69, 70, 74, 75]. PCP perceptions included believing that patients were at low risk [53, 54, 62, 75] and that chlamydia was not a high priority for patients, particularly in rural areas and areas of high deprivation [61, 64, 65, 70]. Gender-related beliefs included a perception that young men did not attend general practice often [61, 66, 69, 74, 75] and that women preferred to see female general practitioners (GPs) for testing which could discourage male practitioners from offering tests [55]. Some also believed that patients prefer to access sexual health services from speciality clinics, and if a patient wanted a test, they would request one [74, 75].

*Beliefs about consequences of offering* [54, 66, 69, 70, 73, 74]. Some PCPs believed offering testing could offend patients by assuming sexual activity or promiscuity. This was consistent with research with young women who felt it important for PCPs to stress that a test offer does not imply their behaviour differs from the norm (relating to social opportunity) but rather was a result of a blanket testing policy [38, 43].

##### Automatic motivation

ᅟ

*Difficult to discuss* [52, 60–62, 64, 66, 68–70, 73, 75]. Some, especially older male PCPs [69], found it difficult to discuss sexual health with patients due to personal discomfort [52, 60, 64, 66, 68, 70]. This was particularly a concern in consultations with male patients [61, 62, 68] and in consultations unrelated to sexual health [64, 67, 68, 70, 73, 75]. This may relate to perceptions that women were more accustomed to sexual health-related discussions with PCPs due to reproductive health appointments (e.g., contraception, cervical screening) [61].

##### Social opportunity

ᅟ

*Practice social norms* [66, 73]. Working in a practice where chlamydia testing or screening was not the norm [66] and lack of support from colleagues [73] could discourage PCPs.

*Provider-patient relationship* [48, 55, 58, 59, 66, 74]. Some PCPs were unwilling to introduce sexual health during new patient checks in case it affected the doctor-patient relationship. PCPs expressed concern about privacy and confidentiality [74, 75], particularly in rural areas where they will likely know their patients socially [58, 59]. They were also reluctant to raise testing if a parent was present in the consultation [66] or if the patient’s family was known to staff [74]; which is supported by research with patients [40]. Patient cultural and religious factors could also act as a barrier to testing [48, 50, 55, 60].

#### Facilitators

##### Physical capability

ᅟ

*Skills-based training* [56, 59, 60, 62, 64, 65, 67, 70, 73–75]. PCPs were willing to conduct testing if trained [66] and GPs with training in STIs were more likely to offer testing [56, 65]. Training and the use of scripts increased confidence (reflective motivation facilitator) [59, 62, 75]. Training should be short but regular and mandatory [74] and should focus on how to make offers without increasing consultation time [64, 70, 75], managing testing and treatment [60, 75], preserving confidentiality [73], and dealing with patients under 16 years of age [73].

##### Psychological capability

ᅟ

*Increasing knowledge, awareness, and education* [54–56, 59, 62, 64, 65, 67, 70, 73, 75]. GPs with postgraduate education in STIs were more willing to offer testing to men as well as indicating greater knowledge of the need to offer to both men and women [56]. Education should focus on the nature of chlamydia infection [64], benefits of testing [73], who and when to test [64], how to manage partners [55, 75], wider sexual health issues [62], and stress the positive views of patients towards testing [70]. Older male PCPs may need specific education due to the age gap and cultural barriers between them and the target population [67]. Providing education enables PCPs to answer questions and increases self-confidence regarding testing [59]. PCP awareness could be increased through campaigns with posters and leaflets [54] and the introduction of national target-based reward and incentive programmes (such as the Quality and Outcomes Framework in the UK) [62, 66].

##### Reflective motivation

ᅟ

*Increase confidence* [59, 62, 70]. Skills-based training and increasing psychological knowledge could facilitate testing by increasing confidence in offering tests. This could also help raise self-esteem and feelings of empowerment through helping PCPs (PNs in particular) realise they can make a difference with their provision of testing [59].

##### Physical opportunity

ᅟ

*Mode of testing* [55, 62, 64, 73, 75]. Self-taken and non-invasive sampling is more acceptable to patients [40, 44] and reduces workload for PCPs [62], thereby facilitating testing.

##### Social opportunity

ᅟ

*Consultation social context* [45, 55, 56, 61, 64, 67, 70, 73]. PCPs found it easier to raise chlamydia testing in the context of sexual and reproductive health consultations, given the reasons previously discussed. Patients reinforced the acceptability of this approach [43, 44].

### Service level factors

#### Barriers

##### Physical capability

ᅟ

*Practice nurse involvement* [58–60, 71]. There were concerns about funding and remuneration for the expansion of PN roles, increases in workload, and time constraints within consultations [58]. Some PN felt a lack of support from GPs [58]. Linking to social opportunity and automatic motivation, some PN in rural areas felt that patients may have privacy concerns [58, 59].

##### Psychological capability

ᅟ

*Lack of testing guidance* [64]. Many of the barriers faced by practice staff, such as lack of knowledge and discomfort in discussing testing with patients, relate to lack of guidance, for example, clarity on when and how to test asymptomatic patients.

*Lack of knowledge, education, and general awareness* [55, 60, 63–66, 71]. Within the practice, knowledge gaps included the epidemiology and presentation of chlamydia, evidence for advantages of testing, populations at-risk, specimen collection, and appropriate treatment.

##### Reflective motivation

ᅟ

*Targets set too high* [70, 74]. Testing targets perceived to be unachievable can result in a practice disengaging from testing, and realistic targets need to be set, reflecting the area (e.g., rural, urban) in which a practice is located.

*Testing policy: new patients’ health checks* [64]. Some GPs expressed reluctance to bring up chlamydia or even sexual health during new patient health checks, as they believed it could hinder the development of the doctor-patient relationship (social opportunity) and felt patients would not want information about chlamydia on their health record.

##### Automatic motivation

ᅟ

*Testing policy: based on behaviour* [41, 48]. Testing policies which are based on sexual behaviour had the potential to cause offence to patients, made PCPs feel uncomfortable, and were felt to evoke embarrassment and shame for the patient [48].

##### Physical opportunity

ᅟ

*Written invitations* [61, 67]. Written invitations to test had disappointing results and could reduce engagement if patients were embarrassed by receiving a letter and the risk of others seeing it. Invitations should highlight that all individuals in their age group are being offered a test (i.e., blanket testing policy) [61].

*Service cost to patient* [37, 39, 47, 60]. Young people and PCP mentioned that the cost of testing for the patient was a barrier to testing.

*Absence of systems to record test offers* [66, 69]. A lack of systematic approaches to call and recall for testing made it difficult to audit testing offers and uptake. Implementing a policy of offering a test every time a young person attends risks offence [69].

*Lack of support for partner notification* [55, 60, 62–64, 73]. Many PCPs felt they did not have the necessary support for partner notification and expressed uncertainty about how it worked, indicating a need for skills-based training [63, 73].

##### Social opportunity

ᅟ

*Testing policy: women only* [37, 38, 69].Testing policies focussing exclusively on women miss the opportunity to test men and reduce men’s responsibility for sexual health [69]. This exacerbates stigma as it associates women with chlamydia and presumed promiscuity [37].

*Cultural norms* [66, 73]. Cultural norms within a practice were discussed in two studies and an environment where testing is not a high priority was seen as a deterrent.

#### Facilitators

##### Physical capability

ᅟ

*Practice nurse involvement* [55, 58, 59, 63, 66, 69, 71]. The involvement of PNs was viewed positively by both GPs and PNs. PNs expressed willingness for increased involvement in testing and management. They are often the first PCP to see patients (particularly young people); young people feel more comfortable speaking to a PN; and, PNs have more time to spend with patients. This approach could reduce the time and workload constraints for GPs and was also viewed favourably by patients [38, 39]. Training and education would be required to enable this facilitator [59].

##### Psychological capability

ᅟ

*Prompts and reminders* [55, 66, 67, 70, 75]. Computer prompts/reminders facilitate testing but rely on practices putting systems in place and recognising the risk of prompt fatigue [66].

*Testing guidelines* [55, 60]. In one Australian study, over 90% of GPs indicated that they would be likely to increase testing if national testing guidelines were introduced and enforced [55]. In some cases, increasing awareness of existing guidelines could facilitate testing [60].

##### Automatic motivation

ᅟ

*Reward and incentive programmes* [51, 55, 61, 62, 66–69, 74, 75]. Evidence on the acceptability and impact of such programmes was lacking. Some PCPs interviewed suggested that having an incentive programme would help testing become a priority [66] and other PCPs indicated that they would increase testing if offered incentive payments for each test performed [55]. This was consistent with a drop in testing when previously offered practice incentives were removed [61]. In contrast, a small financial incentive alone did not increase chlamydia testing in another study [51] while PCP elsewhere did not support financial incentives as they believed they should be providing testing as part of their clinical governance service provision without extra payment [67] and questioned how incentives could be justified if the testing does not have to involve a PCP [62]. The need to pair incentives reminder and feedback systems was emphasised [51]. However, there remains uncertainty as to how to offer any incentive, for example, how much should it be and should it be offered to the practice or the PCP [51].

*Feedback on efforts* [70, 74, 75]. Regular feedback helped personally motivate PCPs and facilitate the embedding of chlamydia testing into general practice. Feedback should be sustained [74] and focus on testing rates and the numbers of tests performed [70, 75].

##### Physical opportunity

ᅟ

*Promotional materials* [54, 63, 70, 74, 75]. Posters and leaflets in waiting rooms or handed out by reception were cited as effective tools for encouraging patients to ask for tests. In one chlamydia testing pilot programme, PCPs identified patient-targeted posters and leaflets as being vital to the pilot’s success. However, it was also pointed out that promotional materials may lose their impact if left on display too long [54].

*Testing policy: inclusion in other consultations* [48, 66, 67]. Offering testing as part of other consultations (e.g., new patients’ health checks, travel vaccination consultations) was considered an enabler to test new patients in the target population and those with who may rarely visit a GP [66]. This approach could also help normalise testing (social opportunity).

*System to record offers*, [66]. The introduction of a system which records testing offers and uptake would facilitate testing, and also prevent multiple offers, which some PCPs feared would lead to offence or irritation.

*Simplified laboratory systems* [59, 62, 66, 68]. Simplified request forms and processes for data feedback from pathology providers was supported.

*Support for partner notification* [62, 64, 69]. Having support and pathways for partner notification may encourage more PCPs to offer testing. Some believed responsibility for partner notification should lie with sexual health clinics [69], and any increase in testing should be accompanied by an increase in staffing [64].

##### Social opportunity

ᅟ

*Testing policy: blanket testing* [38, 48]. Young people felt it important for PCPs to stress that a test offer does not signify their behaviour deviates significantly from the norm (relating to social opportunity) but rather was a result of a blanket testing policy.

### Cross-cutting factors

Three over-arching factors were identified which transcend patient, provider, service levels, and span multiple COM-B subcomponents.

#### Barriers

##### Physical opportunity

ᅟ

*Time constraints*. Patients described consultations as often “rushed” and were aware of the limited time that PCPs have [40, 41, 48]. PCPs [51, 52, 55, 57–64, 66, 68–75] reported that consultation length was insufficient to allow testing, in addition to discussing the primary consultation reason and other priority issues. Testing requires time to discuss sexual health, gain permission, and raise partner notification [52, 68].

#### Facilitators

##### Social opportunity

ᅟ

*Normalisation*. Normalising chlamydia testing for patients [38, 41–43, 61], PCPs [54, 61, 66, 67, 70, 74], and at service level [52, 54, 55, 61, 63, 66, 67, 70] was raised as a way of destigmatising chlamydia infection and facilitating testing. Services in which testing was part of everyday practice (e.g., new patient checks, travel vaccinations, or young people’s clinics) reported high levels of testing [66, 67]. Several strategies were proposed. First, framing chlamydia as a public health issue would allow more open discussion. Second, avoiding requests for the patient to provide a detailed sexual history (particularly partner numbers) when testing, which would also counteract the barrier of embarrassment. Third, blanket testing policies in which all young people are offered a test, which could also reduce automatic motivation barriers for patients (fear of judgement, embarrassment, and shame) and staff should also make this policy clear when offering tests to patients [70, 74] or sending reminder letters [61]. Fourth, education campaigns for patients and the general public [38, 43]. Fifth, promotion and discussion of testing at staff practice meetings [66]. Sixth, fostering a culture of shared learning by talking with staff about difficulties, team huddles prior to clinics, and regular reminders [63]. A flexible approach to testing is also important; practices should adopt a testing policy that suits their patients, practice layout, staffing, and opening times [67].

#### Barrier/facilitator

##### Physical capability and physical opportunity

ᅟ

*Receptionist involvement* [38, 40, 41, 44, 48, 66, 67, 69, 70, 74, 75]. The involvement of reception staff could facilitate testing by reducing the barrier of workload and time constraints. In one study, practices offered patients self-testing kits without a consultation to save time [66]. In another study, it was estimated that testing could add 10 min to a consultation, which could be reduced to 2 or 3 min if patients were provided with the testing form and leaflet at reception [69]. However, receptionists lack medical training (psychological capability) and so may be ill-equipped to answer patient questions regarding testing [44, 66, 67, 69, 70]. Young people [38, 40, 41, 44, 48, 70] and PCP [74, 75] deemed the reception area to be an unacceptable location to offer information about chlamydia due to patient privacy concerns (social opportunity).

## Discussion

This is the first systematic review to conduct a theoretical analysis of barriers and facilitators to chlamydia testing for young people and PCP in general practice. Building on previous work in this field, this review demonstrates considerable overlap between perceptions of young people and PCP on the barriers and facilitators to chlamydia testing across patient, provider, and service levels. Both groups emphasised the potential of the chlamydia testing policies (e.g., testing based on patient behaviour; women-only testing) to imply judgements about sexual behaviour and identity, particularly through women-only testing or when sexual history was asked. This had particular resonance for women who were more often offered testing in a context where staff described widespread reluctance to initiate sexual health conversations with men. Patients and staff agreed on the need to offer tests within a context that fully addressed concerns including the potential stigma of chlamydia testing. The need to normalise test offers as universal, and embed it in routines, was emphasised by both groups as key to minimising the stigma of feeling judged. Concerns about privacy were also emphasised by both groups, particularly where the reception area and staff were involved in implementing testing. This was seen as a place where young people felt exposed, particularly in small town or rural settings. Both groups experienced the allocated time for consultations as a competing pressure, and PCPs struggled to reconcile the need to discuss the relevance of testing to young people given the workload this would create, whatever the model for test offer.

While most themes could be categorised with the COM-B Model, some did not fit neatly within one subcomponent. This mirrors the hypothesised relationships between components of the model (Fig. 1); opportunity and capability can influence motivation, while behaviour can alter capability, motivation, and opportunity. For example, PCPs who lacked training (physical capability) and knowledge regarding chlamydia testing (psychological capability) were less confident in conducting tests (beliefs about capability—reflective motivation). Forgetfulness (psychological capability) related to lack of a reminder system (physical opportunity). Patient’s perceived risk (reflective motivation) was mediated by psychological capability through awareness of chlamydia. Furthermore, we categorised the emotions of embarrassment and shame under automatic motivation. However, these closely link to the social opportunity component as these feelings result from the comparison of the self to social standards. The intersections across subcomponents reflect the complexity of chlamydia testing behaviour.

### Limitations

PCP views of what young people may feel about chlamydia testing in the general practice setting provide some clues about barriers and facilitators to implementation. However, these are expressed in a setting and over a period where chlamydia and other STI testing practices and rates remain highly variable [78]. PCP comments may be offered to justify or rationalise a *status quo*, and suggested facilitators or barriers may or may not be correctly identified. Generally, studies did not report the demographic features or testing patterns of the practices in which PCP worked, which would have helped contextualise the comments of staff.

Young people participating in these studies may or may not have experienced an offer of chlamydia testing, and if they have, it may have been in any setting. While indicative, their feelings at interview may or may not represent what would actually happen if offered a test at their GP surgery, and again, the studies available do not allow us to interrogate their actual experience.

### Implications for policy and practice

This review has built on previous literature by highlighting the complex determinants of chlamydia testing. Across all three levels, multiple barriers and facilitators were identified relating to psychological capability and physical and social opportunity. Given the nature of the studies included (mostly cross-sectional), we cannot state which component represents the most important influence on chlamydia testing. To increase testing, we should focus on targeting multiple factors, specifically (1) normalisation, (2) communication, (3) infection-specific information and education, and (4) mode of testing. Normalisation and integration into routine appears to be an influential facilitator with testing integrated into everyday practice and could be reinforced by external sources, such as national guidelines and reward and incentive programmes based on outcomes. Second, good communication in the interaction between the PCP and patient is essential. Offers need to be framed appropriately; emphasising the offer is universal while sexual history taking must be approached with caution since it may undermine testing. Third, educational and awareness interventions for young people should focus on infection-specific information (i.e., chlamydia’s long-term impact on fertility and its asymptomatic nature). Finally, regarding mode of testing, small modifications (such as the use of urine testing) have the ability to simultaneously reduce multiple barriers such as time, workload, and stigma. It is clear that in order to increase chlamydia testing in primary care and reduce the transmission chain in the population, a multifaceted theory- and evidence-based approach is needed.

## Conclusions

Unlike previous reviews, we took a multi-level theoretically informed approach to synthesise data addressing barriers and facilitators to chlamydia testing in general practice. Through the application of COM-B, a coherent framework for explaining chlamydia testing has been developed. This review is only the first step towards developing theory- and evidence-based interventions to increase chlamydia testing in general practice. Future research should identify the intervention types and behaviour change techniques which would be suitable to address the factors identified to improve implementation of chlamydia testing in general practice and reduce the transmission chain in the population.

## Additional files

Additional file 1: PRISMA Checklist. (PDF 23 kb)

Additional file 2: Search Strategy for MEDLINE. (PDF 65 kb)

Additional file 3: Findings of included studies with primary care professionals. (PDF 246 kb)

Additional file 4: Findings of included studies with young people. (PDF 221 kb)

Additional file 5: Illustrative quotes for each theme. (PDF 161 kb)

## Abbreviations

COM-B model: Capability, Opportunity, Motivation, Behaviour Model
GP: General practitioner
NAATs: Nucleic acid amplification tests
NCSP: National Chlamydia Screening Programme
PCP: Primary care professional
PN: Practice nurse
PRISMA: Preferred Reporting Items for Systematic Reviews and Meta-Analyses
RCT: Randomised controlled trial
STI: Sexually transmitted infection
