# Supplementary material for: Application of the COM-B model to barriers and facilitators to chlamydia testing in general practice for young people and primary care practitioners: a systematic review
Source: Implement Sci. 2018 Oct 22;13:130. doi: 10.1186/s13012-018-0821-y (PMC6196559; doi:10.1186/s13012-018-0821-y)
Supplement: Supplementary file 3 — Findings of included studies with primary care professionals. (PDF 246 kb) [file 13012_2018_821_MOESM3_ESM.pdf]

Additional File 3. Findings of included studies with primary care professionals, by level (young people, provider, service), and sub-component (COM-B)

Heading abbreviations: COM-B = capability, opportunity, motivation, behaviour; Y = young people; P = provider level; S =service level; PhC = physical capability; PsC = psychological capability; RM = reflective motivation; AM = automatic motivation; PO = physical opportunity; SO = social opportunity

**Source**	**Barriers**	**Level**			**COM-B Subcomponent**						**Facilitators**	**Level**			**COM-B Subcomponent**					
		**Y**	**P**	**S**	**PhC**	**PsC**	**RM**	**AM**	**PO**	**SO**		**Y**	**P**	**S**	**PhC**	**PsC**	**RM**	**AM**	**PO**	**SO**
Allison et al. (2017)	Assumptions about patients: if a patient wanted to be tested for chlamydia, they would just pick up a self-screening kit		✓				✓				Regular updates circulated to all staff - initially staff were motivated and focussed, but as time passed, was not sustained		✓	✓				✓		
	Assumptions about patients: preference for receiving sexual health advice from services other than general practices		✓				✓				Well displayed posters			✓					✓	
	Assumptions about patients: concerns regarding lack of confidentiality in general practice, if patient's family is known to practice staff		✓				✓				Phrase the offer correctly, emphasise the routine, non-judgemental nature of the offer	✓	✓	✓		✓				✓
	Perception that young men do not attend frequently		✓				✓				Financial incentives could make a small difference			✓				✓		
	Perception that patients are offended and feel judged if offered in a non- sexual health consultation		✓				✓				Short but regular training slots, mandatory training			✓	✓					
	Some reception staff felt uncomfortable handing out invitation cards to patients		✓	✓				✓												
	Targets set to gain financial incentives were unrealistically high			✓			✓													
	Lack of time and competing priorities (patients attending with more than one issue to discuss)	✓	✓	✓					✓											
	Lack of privacy in the reception area			✓						✓										
Bilardi et al. (2010)	Lack of provider education and awareness		✓	✓		✓					Financial incentives for GPs (41%)			✓				✓		
	Forgetting: Many initially increased testing at the start of the trial, but over time did not remember to offer testing, and/or		✓			✓					Patient education or awareness about testing (31%)	✓			✓					

	forgot about the incentive payment																		
											Computer prompts or reminders to test (26%)			✓		✓				
Bilardi et al. (2009)	Lack of time		✓	✓	✓															
	Discomfort with raising the issue of testing		✓					✓												
	Difficulty in remembering to refer patients		✓			✓														
Calamai et al. (2013)	Perception that they did not see too many high-risk patients and therefore did not offer testing		✓				✓													
Freeman et al. (2009)	Many staff thought posters and leaflets would cause offence			✓			✓				Leaflets: Pocket-sized, pictorial, reflect multi-cultural society			✓					✓	✓
	Distribution of leaflets by receptionists was thought to be inappropriate - patients would be offended when being offered a leaflet in a public area	✓		✓				✓		✓	Receptionists in high screening practices proactively gave patients in the at-risk age group a chlamydia leaflet		✓	✓	✓					
	Reason for not offering test - perception that they 'did not see too many high-risk patients'		✓				✓				Mobile phone texts rather than sending letters to patients' homes			✓					✓	
											National television advertising			✓	✓					✓
											Information on practice websites			✓						✓
Hocking et al. (2008)	Lack of time		✓	✓	✓						Financial incentives for GPs, incentive payment available for each chlamydia test performed			✓				✓		
	Lack of knowledge of GPs and the public about chlamydia	✓	✓	✓		✓					Funding for practice nurses			✓					✓	
	Patient embarrassment	✓						✓			Education for GPs, PNs and the general public	✓	✓	✓		✓				
	Religious and cultural issues, need to be addressed in a sensitive and culturally appropriate way	✓		✓						✓	National screening guidelines			✓		✓				
	Women prefer to see a female GP for chlamydia testing		✓				✓				Introduction of a formal screening program with associated recall mechanisms			✓		✓			✓	
	Lack of a formal recall/reminder system for chlamydia testing			✓					✓		Greater support for contact tracing			✓					✓	
	Lack of support for contact tracing			✓					✓		Destigmatization	✓								✓
											Majority of GPs consider that it is important to be able to test			✓					✓	

											urines rather than swabs									
Khan et al. (2006)											GPs who had postgraduate training in STIs had double the odds of offering testing to young men and to female patients at the time of smear		✓	✓	✓	✓				
Lorch et al. (2013)	Patients' lack of chlamydia knowledge (69%)	✓				✓					Providers require additional training or skills to manage testing and treatment of chlamydia (89%)		✓	✓	✓	✓				
	Time constraints during consultations (53%)		✓	✓					✓											
	Lack of a formal chlamydia test recall/reminder system (46%)			✓					✓											
	Lack of support for partner notification (46%)			✓					✓											
	Patient religion/ethnicity (33%)	✓								✓										
	Lack of support for PNs (26%)			✓					✓											
	Difficulty talking with clients about sexual health (21%)		✓				✓													
	Cost of testing to client (21%)	✓		✓					✓											
	The chance of patients getting a false positive result (10%)		✓				✓													
	Concerns about over servicing (4%)		✓	✓					✓											
Lorch et al. (2016)	Time or workload constraints		✓	✓					✓											
	Difficulty offering or discussing testing in a non-sexual health consultation		✓				✓													
Lorch et al. (2015a)	Concerns related to funding for PN: concerns about how their additional activities would be renumerated			✓					✓		GPs felt that PNs had more time for patient education and advice		✓	✓					✓	
	PN workload: makes it difficult for them to expand their role			✓					✓		GPs felt that patients would find PNs easier to talk to and less intimidating than GPs	✓	✓				✓			✓
	PN role: issues of patient privacy and confidentiality related to living in a "small town" privacy/ confidentiality	✓	✓	✓						✓	PN role: GPs could benefit through a reduction in their workload		✓	✓					✓	
Lorch et al. (2015b)	Time and workload constraints		✓	✓					✓		Perceived that patients would feel more comfortable engaging with a PN rather than a GP	✓		✓			✓			

	Concerns around privacy in the “small town” rural settings of the general practices			✓						✓	Involvement of PN would alleviate doctors’ workloads			✓					✓	
	PN role: GP negative attitudes towards PNs -role conflict and handing over power		✓	✓			✓				Provision of training and education		✓	✓	✓	✓				
	Issues with funding for PNs’ work			✓					✓		Changes to pathology ordering processes				✓				✓	
	Pathology ordering			✓					✓		Role expansion for PN could lead to greater job satisfaction and empowerment		✓				✓	✓		
Lorimer et al. (2014)	Perception that young men did not attend very often		✓				✓				Internet-based screening approach	✓	✓	✓			✓		✓	
	Screening linked to the nature of the patient-led consultation - easier to raise issues of sexual health with patients when attending for related issue (e.g., cervical screening)		✓				✓				Normalisation, could be successful if young people considered testing as a normal thing to do, could be assisted by a nation-wide marketing campaign	✓								✓
	Reluctance to initiate conversations around sexual health with men - encounters seen as ‘difficult’, ‘awkward’ and ‘challenging’		✓				✓													
	Not confident that chlamydia was a high priority for their patients		✓				✓													
	Fail to raise the issue of screening with men, particularly in areas of higher deprivation		✓				✓													
	Uneasy with ‘unsolicited health care intervention’ and with making health promotion ‘leaps’		✓				✓													
	Time pressure		✓	✓					✓											
	Chlamydia screening higher in practice when payments were offered but reduced once there was no longer a financial incentive for the practice			✓				✓												
	Socio-cultural barriers, perceptions among young people that chlamydia is a ‘woman’s disease’, associated with infertility and promiscuous	✓	✓				✓			✓										

	women																			
	Believed that such young people fear the stigma of attending for an STI test where they can be seen and identified as promiscuous	✓	✓				✓			✓										
	Believed women more likely to beat ease with screening offers, given their experiences of cervical screening and contraception appointments		✓				✓													
Ma & Clarke (2005)	Financial remuneration - extra workload due to screening but others questioned how remuneration could be justified if the screening test is free			✓					✓		Reduce the need to talk about sex, i.e., "blanket" screening policy	✓	✓	✓						✓
	Time pressure		✓	✓					✓		Ease of administering a test: urine tests for men and self-taken vulval-vaginal swabs for women might increase acceptance for screening and would lessen impact on clinicians’ workload	✓	✓	✓					✓	✓
	Concerned about the volume of partner notification from the screening			✓					✓		Awareness campaign for public and professionals to de-stigmatise	✓	✓	✓		✓				✓
	Urine tests for men and self-taken vulval-vaginal swabs are more expensive and technically difficult - not all of the local laboratories can process them			✓					✓		Training to be confident with raising screening opportunistically		✓	✓	✓		✓			
											Multidisciplinary training events on topics such as confidentiality, under-16s and wider sexual health issues		✓	✓		✓				
McKernon & Azariah (2013)	Clinical difficulties - deciding on treatment (20%)		✓			✓					Customisation of the practice management software to support individual staff member’s testing knowledge and behaviours		✓							
	Skills-based difficulties - how to word the offer to test (28%)		✓		✓						Patient-oriented promotions (posters and pamphlets)			✓						✓
	Contextual difficulties - preserving confidentiality (46%)		✓		✓					✓	Fostering a culture of shared learning (such as by talking with		✓	✓		✓				✓

											other staff about difficulties and learnings, team huddles prior to clinics, and regular reminders)									
	Operational difficulties - completing tasks within the appointment time (32%)		✓						✓		Having better privacy at reception and triage areas	✓		✓						✓
											Make chlamydia testing a key performance indicator for the PHO			✓				✓	✓	
											Expand screening to other settings, such as schools, universities and workplaces			✓					✓	✓
											Having a screening service run by nurses			✓					✓	
											Having more time for screening consistently			✓					✓	
McNulty et al. (2004a)	Perceived lack of evidence of the benefits of chlamydia testing		✓			✓	✓				Test at smear consultation & send patient leaflets pre-appointment			✓					✓	
	Lack of knowledge of when and how to take specimens		✓		✓	✓					Specific clinic for chlamydia tests: properly resourced drop-in clinic			✓					✓	
	Lack of time		✓	✓					✓		Guidance and education for healthcare staff and patients: particularly for asymptomatic patients; short and clear guidance re background information, and when and how to test for genital chlamydia	✓	✓	✓	✓	✓				
	Discussing chlamydia in consultations unrelated to sexual health: thought that it would upset patients		✓				✓				Non-invasive specimens - urine sampling	✓		✓					✓	
	Inclusion in new patients' health checks: thought that it would hinder development of the doctor–patient relationship		✓				✓				Develop skills to discuss sexual health		✓			✓				
	Concerns about discussing chlamydia at cervical smear examination: lack of confidence about the information they should be giving to the patient		✓			✓	✓				Clear lines of responsibility for contact tracing			✓					✓	
	Contact tracing		✓	✓					✓		Increase in trained staff in primary care			✓					✓	

	Attitudes to Department of Health screening programme proposals: did not believe the Department of Health had ‘any idea of what really goes on in general practice’ or the ‘extreme pressures GPs were under		✓				✓														
	Not willing to discuss chlamydia in a consultation unrelated to sexual health		✓					✓													
	Lack of guidance			✓					✓												
McNulty et al. (2004b)	Lack of confidence in managing men with genitourinary symptoms		✓				✓				Incorporating testing into routine practice (e.g., vaginal swabs)			✓					✓	
	Lack of communication with sexual health clinics			✓					✓		Having a GP staff member who had an interest in sexual health, had attended continuing education or participated in specialist training in family planning		✓	✓		✓			✓	
	Differing thresholds for testing: low testing practices only considered testing after multiple consultations with symptoms			✓					✓		Availability of patient information leaflets on chlamydia			✓					✓	
	Lack of knowledge around epidemiology and presentation of chlamydia, and accuracy of testing		✓			✓					Awareness of risk of chlamydia among practice population	✓				✓				
	Perception that practice population is at low risk, due to age and rural status		✓				✓													
McNulty et al. (2010)	Lack of support from entire practice team and chlamydia screening had not become the norm		✓	✓						✓	Offer screening at new patient checks			✓					✓	
	Not comfortable raising chlamydia screening: fear it could cause offence and		✓				✓				Normal practice to offer most patients screening (normalisation)			✓						✓
	Insufficient training		✓		✓	✓					Regular practice meetings and ongoing support from NCSP coordinators			✓					✓	✓
	Receptionists were not trained appropriately		✓	✓	✓						Easy availability of screening kits would save time - leave packs in reception area			✓					✓	

	Receptionists did not have sufficient time			✓					✓		Computer templates reminders/alert - but be aware of prompt fatigue			✓					✓	
	Poor relationship with NCSP coordinator			✓						✓	Simplified request forms			✓					✓	
	Confidentiality issues: reception area not confidential enough for receptionists to discuss chlamydia screening with patients			✓						✓	Increase staff awareness of targets and QOF: if chlamydia was included in the QOF priorities, they would consider it more important and develop a much more systematic approach to screening		✓	✓		✓			✓	
	Time pressures: reluctant to extend consultations			✓					✓		Nurse led approach			✓					✓	
	Forgetfulness			✓	✓						Would be easier if patients were made more aware of screening through increased use of leaflets and posters	✓	✓	✓		✓			✓	
	Recording offers of screens: no computer Read codes to record chlamydia screening was offered chlamydia screening and if the patient had agreed or declined, therefore it would be difficult to audit screening offers and uptake			✓					✓		Time pressures: easy availability of screening kits would save time; leave packs in reception area			✓					✓	
McNulty et al. (2008)	Written invitations for chlamydia screening to the target population - disappointing results unless it was combined with another high-profile public health interventionsuch as the mumps immunization programme			✓					✓		Having a practice champion, who drives the screening process forward and maintains the motivation of other practice staff			✓			✓		✓	✓
	Perceived increase in workload		✓				✓				Normalise screening - part of everyday practice - at all consultations all at-risk patients, not just those involving sexual health	✓		✓						✓
	Lack of comfort in discussing sexual health in consultations un-related to sexual health		✓					✓			Computer-generated reminders that flagged up patients in the target age group (time-saving method)			✓					✓	
											Patient-completed screening kits - offered either by receptionists when patients booked in, or by health-care staff after a	✓		✓					✓	

											consultation, and then picked up by patients in the clinical and/or reception area (time-saving method)									
											Screening when new patients register			✓					✓	
											Chlamydia screening sustained through frequent reminders, newsletters containing chlamydia screening rates, and advertising to the ‘at-risk’ population from the screening team			✓					✓	
											Programme needs to have a high national and local profile through: support from PCTs (CCGs) and local medical committees			✓					✓	
											promotional materials/media campaign for target population			✓		✓				✓
											A flexible approach to the screening process: allow practices to adopt a screening policy that suited their practice layout, staffing, surgery times and population			✓					✓	
											Targets should be set for practices to encourage screening			✓				✓		
											Some supported incentive/payment - others did not, should be providing screening as part of their clinical governance service provision without extra payment			✓				✓		
											Chlamydia screening as a core requirement of the General Medical Services contract			✓					✓	
											Rural practices: providing chlamydia screening with contraception advice			✓					✓	
											Ongoing training of staff: Older male GPs need specific education due to age gap and cultural barriers between them		✓	✓		✓				

											and the target population									
											Receptionists, if involved, need specific education package		✓			✓				
McNulty et al. (2017)	Difficult to ask about sexual health in an unrelated consultation		✓					✓			Need for an update on sexual health and chlamydia, how to test, treatment and partner notification		✓		✓	✓				
	Perceived that not many young patients attended, especially young men.		✓				✓				Posters, invitation cards, leaflets and information on practice websites would facilitate testing	✓		✓		✓			✓	
	Perception that patients were not at risk		✓				✓				Time barrier could be decreased if GP staff were taught how to make the offer using short scripts of common consultations		✓		✓					
	Perception that patients preferred to seek sexual health services elsewhere		✓				✓				Computer prompts or templates			✓		✓				
	Believed that patients would be concerned about confidentiality		✓				✓				Enthusiastic about self-taken vaginal swabs			✓					✓	
	GP staff perception that patients need to request a test		✓			✓	✓				Patients should do the test in practice to increase number of tests returned	✓		✓					✓	
	Time and pressures of other waiting patients		✓	✓					✓		Receiving feedback on testing rates			✓				✓		
	Forgetting		✓			✓					Involving receptionists			✓					✓	
	Limited funding for testing meant staff were less likely to offer tests in comparison to paid for public health interventions		✓					✓												
	Many GP staff were not completely aware of the exact specimen collection procedures		✓		✓	✓														
	Concern about patients actually returning the self-sample kits	✓	✓				✓													
Merritt et al. (2007)	Insufficient time during a standard consultation. Considered particularly time consuming when dealing with patients with limited or no previous knowledge of chlamydia, and pre-test counselling was considered	✓	✓	✓		✓			✓		A performance-related financial incentive for GPs (similar to those offered for childhood immunisation in Australia			✓				✓		

	enormously time intensive																			
	Forgetting to consider testing in the target group		✓			✓					Increased community awareness of chlamydia	✓		✓		✓				
	Difficulty in raising the concept of chlamydia testing during unrelated consultations, particularly for males and patients who were very ill		✓					✓		✓	A simplified process for data feedback from pathology providers to monitor testing performance for each practice or practitioner			✓					✓	
Perkins et al. (2003)	GPs reluctant to manage test results because of the potential impact on their work-load		✓						✓		Giving out forms and leaflets at reception saved time			✓					✓	
	Older male colleagues were not comfortable with sexual health work		✓					✓			PN could take on screening responsibilities			✓					✓	
	No consensus regarding adequacy of payment among the GPs			✓				✓			PN believed that young women were more comfortable with them screening than GP		✓				✓			
	Receptionists - felt ill equipped for sexual health discussions (no medical training) and unsuitably located (in public space) for privacy and confidentiality	✓	✓		✓	✓				✓	Contact tracing and partner notification - believe this is better undertaken by sexual health clinics			✓					✓	
	Potential for embarrassment if parent or partner present	✓						✓												
	Focus exclusively on women - potential to cause offence and damage self-esteem by suggesting sexual activity or promiscuity; reduces men's responsibility for sexual and reproductive health	✓		✓			✓	✓												
	Potential for test to be offered more than once - could cause offence to patient	✓		✓				✓												
	Belief that men rarely attended, little experience of any sexual health work with young men, and would be reticent about offering them screening		✓				✓													
Ricketts et al. (2016)	Forgetting to offer chlamydia screen		✓			✓					Using computer prompts and posters, and identify individual to take this forward			✓					✓	
	Not wishing to offer chlamydia		✓					✓			Staff changing the way they		✓	✓	✓				✓	

	screening in non-sexual health consultations										offer a chlamydia screen by stating that they are testing everyone aged between 15 and 24, and having a general prompt (and a narrated phrase) for all ages									
	When sexual health is a low priority in the general practice - participants' own views were a barrier to increasing chlamydia screening in their practice		✓							✓	Having a designated general practice lead to drive screening and send around reminders and forward newsletters to all staff			✓					✓	
	Saw it as not always appropriate to raise chlamydia screening; if a parent was present, if the patient was not consulting about sexual health, or if it didn't 'feel' right		✓				✓	✓		✓	Use scripts; increases confidence and fluency in making the offer		✓				✓			
	Concerned that patients would be irritated by the offer		✓					✓			Posters to refer to, kits easily accessible and invitation cards available to give to patients			✓					✓	
	Perception of time involved in offering chlamydia screen, and monitoring screening rates		✓				✓				Audits as part of professional development of all staff			✓				✓	✓	
	Patients not returning the chlamydia test	✓				✓					Feeding back that other general practice staff ask the patient to do the test prior to leaving the general practice, and this is what patients want too	✓	✓	✓		✓			✓	
	Ongoing perception that patients may be irritated if they are offered screening		✓				✓				Stress patients views in the training, lead to facilitate ongoing discussions in general practice around feedback from patients	✓				✓				
	Lack of privacy in reception area to give out invitation cards			✓						✓	Offer should be made to all in age group so not seen as judgemental by staff		✓							✓
	Targets being too high for general practices with very low screening rates			✓				✓			Teach receptionists to use script		✓	✓	✓					
											Realistic screening rates for individual general practices with numbers and actions needed to attain them			✓				✓		
Robertson &	Lack of confidence		✓				✓				Would do if funded			✓				✓		

Williams (2005)	Lack of knowledge (how to use swabs)		✓	✓		✓					Would undertake testing if trained		✓	✓	✓	✓				
	Lack of training		✓	✓	✓															
	Lack of time		✓	✓					✓											
Senok et al. (2005)	How to change the focus of the consultation to chlamydia testing		✓		✓															
	Felt it was inappropriate (especially if consultation involved a mental health problem)		✓				✓			✓										
	Concerns about the time required to raise the issue of chlamydia		✓						✓											
	Postal screening - confidentiality of testing kit recipient	✓					✓			✓										
	Postal screening - test kits were bulky and difficult to post			✓					✓											
Wallace et al. (2012)	Additional time in consultation		✓	✓					✓		Surgery signing up as a whole			✓					✓	✓
	Patient attending with parents	✓	✓				✓	✓			Training on the benefits of screening		✓	✓		✓				
	Comprehension barriers	✓				✓					Knowledge about treatment		✓	✓		✓				
	Patient presents with an issue unrelated to sexual health		✓				✓				Raising awareness via well-displayed posters and leaflets	✓		✓		✓				
	Language barriers	✓				✓					Knowing how to deal with a positive result		✓			✓				
	Problems around contact tracing			✓					✓		Consultation skills		✓		✓					
	Lack of time in consultations		✓	✓					✓		Patient presents with a sexual health issue	✓	✓				✓			
	Lack of support by colleagues			✓						✓	Patient attending for cervical screening	✓	✓				✓			
	Patients being unwilling to talk about STIs	✓					✓				Patient attending for contraceptive pill check	✓	✓				✓			
											Patient attending to obtain contraception	✓	✓				✓			

Note: CCG = clinical commissioning group; GP = general practitioner; NCSP = National Chlamydia Screening Programme; PCT = primary care trust; PN = practice nurse; STI = sexually transmitted infection; QOF = quality and outcomes framework
