# Supplementary material for: Application of the COM-B model to barriers and facilitators to chlamydia testing in general practice for young people and primary care practitioners: a systematic review
Source: Implement Sci. 2018 Oct 22;13:130. doi: 10.1186/s13012-018-0821-y (PMC6196559; doi:10.1186/s13012-018-0821-y)
Supplement: Supplementary file 4 — Findings of included studies with young people. (PDF 221 kb) [file 13012_2018_821_MOESM4_ESM.pdf]

Additional File 4. Findings of included studies with young people, by level (young people, provider, service), and sub-component (COM-B)

Heading abbreviations: COM-B = capability, opportunity, motivation, behaviour; Y = young people; P = provider level; S =service level; PhC = physical capability; PsC = psychological capability; RM = reflective motivation; AM = automatic motivation; PO = physical opportunity; SO = social opportunity

**Source**	**Barriers**	**Level**			**COM-B Subcomponent**						**Facilitators**	**Level**			**COM-B Subcomponent**					
		**Y**	**P**	**S**	**PhC**	**PsC**	**RM**	**AM**	**PO**	**SO**		**Y**	**P**	**S**	**PhC**	**PsC**	**RM**	**AM**	**PO**	**SO**
Balfe et al. (2009)	Stigma, embarrassment and shame about the potential social consequences of being known to attend a health service for testing, discredit young adults' identities	✓						✓		✓	Seeing testing as a responsible practice that adults should engage in	✓					✓			
	Embarrassment: the testing process, having to take clothes off in front of healthcare professional (stronger for women)	✓						✓		✓	Moral obligation	✓					✓			
	Shame: had unprotected sex and were ashamed, concerned healthcare provider would judge them and behaviour	✓						✓		✓	Concerns about the damage an infection could do to future fertility	✓					✓			
	No symptoms therefore not urgent	✓					✓				Perceived risk status of respondents' sexual partners	✓					✓			
	Cost	✓		✓					✓		Anxieties about the risk that asymptomatic STIs could affect them without their knowledge	✓					✓			
Balfe et al. (2010)	Testing seen as a threat to young women's identities: anxieties about promiscuity (risk takers) and identity discreditation	✓						✓			Preference for testing offers from younger female healthcare professions, not receptionist	✓		✓					✓	
	Would not accept tests from 'para-professionals' (healthcare professionals who were not doctors nor nurses) who worked in public settings (reception areas).	✓		✓					✓		Test offered in private areas, rather than in more public settings (e.g., reception areas)	✓							✓	
	Offers framed in moral surveillance style, undermined identities were likely to be rejected	✓					✓				More likely to accept if emphasised that testing is a normal practice and is a good, responsible thing to do	✓	✓				✓			
	Stigma of having an infection	✓								✓										
Brugha et al.	Testing located in pharmacies and public non-health care	✓		✓					✓		Most women wanted to be offered test by a female	✓	✓				✓		✓	

(2011)	settings										healthcare professional (65%)									
	Cost	✓		✓					✓		Most wanted to be offered test by a doctor (75%), 50% of males and 80% of would feel comfortable being test by nurses	✓	✓						✓	
											Preference for urine-based test (90%)	✓		✓					✓	
Ewert et al. (2016)	Embarrassment: talking to someone about personal information	✓						✓			Increase education: sexual health education in schools	✓		✓		✓				
	Embarrassment: if others and parents were to find out	✓						✓		✓	Increase awareness: public health campaigns, e.g., TV advertising	✓		✓		✓				✓
Heritage & Jones (2008)	Tests offered by receptionists			✓					✓		Preference for urine samples over self-taken vulval swabs	✓		✓					✓	
	Policy targeting females only			✓						✓										
	Embarrassment and fear	✓						✓												
	Partner tracing caused concern	✓							✓	✓										
	Lack of privacy in reception area	✓		✓					✓	✓										
	Parent being present during consultation	✓								✓										
Hogan et al. (2010)	Embarrassment: to admit to unprotected sex	✓						✓			Raise awareness: marketing campaigns, posters, letters, leaflets and staff talking to the patients about testing		✓	✓		✓				
	Embarrassment: in case they were seen by someone they knew	✓						✓		✓	Characteristic of doctor or nurse: non-judgemental		✓				✓			✓
	Fear of being judged by the staff at the surgery and fear of parents finding out	✓						✓		✓	Preference for providing the sample immediately at the practice rather than home	✓		✓					✓	
	Scared of results: prefer to ignore the issue, easier when no symptoms	✓						✓												
	Time constraints	✓	✓	✓					✓											
Jones et al. (2017)	Staff judgemental attitude and being singled out as ‘needing’ a chlamydia test	✓						✓			Increasing patient awareness of service	✓				✓				
	Embarrassment: having to walk through reception with sample	✓		✓				✓	✓		Mode of testing	✓		✓					✓	
	Inappropriate in an unrelated consultation if the patient is religious - could cause offence	✓								✓	Routine testing offered to all in age group in all consultations	✓		✓				✓	✓	✓

	Time: testing in an unrelated consultation takes time away from their original reason for attending, thereby reducing quality of consultation	✓		✓					✓											
	Forgetting to return home testing kits	✓				✓														
Mac Phail et al. (2017)											Medical provider offered a test		✓						✓	
											Cost - free			✓					✓	
											Annual reminder to test via email (>50%) or text message (40%)			✓					✓	
Mills et al. (2006)	Embarrassment with sexual health issues and associations with promiscuity	✓						✓		✓	Understanding of benefits of testing	✓				✓				
	Participants may not consider themselves at risk	✓					✓													
	Unease with vulvo-vaginal swab	✓		✓					✓											
	Anxiety: in relation to all stages of the testing process, from offer to receiving results	✓						✓												
	Stigma	✓								✓										
Norman sell et al. (2016)	Fear of having identity 'tainted' or 'spoiled' by the need to get tested	✓						✓												
	Fear of positive result	✓						✓												
	Judgemental attitudes of healthcare professionals		✓				✓		✓											
	Perception that GPs lack sufficient expertise in sexual health	✓	✓		✓	✓														
	Patient cultural and religious factors	✓					✓			✓										
Pavlin et al. (2008)	Confronting nature of being tested for chlamydia and fear of the infection	✓						✓			Age-based testing (16-24 years) acceptable and non-discriminatory	✓		✓					✓	
	If diagnosed with chlamydia concerned would be judged and seen as sexually promiscuous, feel embarrassed and ashamed	✓						✓		✓	Introducing chlamydia during a sexual health-related consultation is appropriate	✓	✓						✓	
	Being required to disclose their sexual history	✓		✓						✓	Normalisation: frame as a public health issue that is openly discussed rather than an issue that relates to the behaviour of	✓	✓	✓						✓

											an individual									
											Normalisation: Widespread community education campaign	✓		✓		✓				✓
											No pressure put to provide a sexual history (questions relating to number of partners)	✓	✓							✓
											Did not want to be contacted by mobile text message with results, email more acceptable. Letter to home acceptable if in unmarked envelope. Phone call acceptable. Face-to-face with GP preferred for results	✓		✓					✓	
											Anonymous contact tracing	✓		✓						✓
Pimenta et al. (2003)	Dissatisfaction with receptionist handing out leaflets - compromised confidentiality	✓		✓					✓	✓	Reassurance that are not being singled out as being “sexually active” or “at risk” of having chlamydia	✓	✓					✓		
											Knowing the possible long term impact on fertility	✓				✓				
											Knowing that chlamydia was easily treated	✓				✓				
											Urine sample is more acceptable and less embarrassing than swabs	✓		✓				✓	✓	
Santer et al. (2003)	Perceived lack of ‘at risk’: no symptoms, sexual history, perceived sexual history of partner, perceived prevalence of chlamydia (not common), lack of awareness of chlamydia	✓					✓				Being informed of the risk of infertility, asymptomatic nature, the ease of testing, infection could be treated with antibiotics	✓				✓				
Zakher & Kang (2008)	Perceived low risk (65%)	✓					✓				Perceived personal risk	✓					✓			
	Not comfortable discussing sexual matters with GP (38%)	✓						✓			Preference for urine sample	✓		✓					✓	
	Not seeing chlamydia as a concern personally or within their peer group	✓								✓	Comfortable with GP test every two years as part of routine check	✓		✓					✓	
	Not knowing what was involved or where to go	✓				✓														
	Fear of what others would think	✓						✓		✓										
	Not wanting to know chlamydia status	✓						✓												
	Cost	✓		✓					✓											
