# Supplementary material for: Application of the COM-B model to barriers and facilitators to chlamydia testing in general practice for young people and primary care practitioners: a systematic review
Source: Implement Sci. 2018 Oct 22;13:130. doi: 10.1186/s13012-018-0821-y (PMC6196559; doi:10.1186/s13012-018-0821-y)
Supplement: Supplementary file 5 — Illustrative quotes for each theme. (PDF 161 kb) [file 13012_2018_821_MOESM5_ESM.pdf]

Additional File 5. Illustrative quotes from included studies across levels (patient, provider, and service) and theoretical sub-component

COM-B Sub component	Patient Level		Provider Level		Service Level	
	**Barrier**	**Facilitator Source**	**Barrier**	**Facilitator**	**Barrier**	**Facilitator**
**Physical Capability**			Lack of training and skills *"I don't think sexual history taking is taught well at medical school for a start. I think how to investigate for any sort of STIs are seen as a sort of scary. And many GPs don't have, perhaps don't even realise that a urine sample is good enough"* [55]	Skills-based training *"I think probably tiny amounts of training on a on a frequent basis…may be better than a big training session which nobody has got time for… I think small but regular training inputs are more effective"* [74]	Receptionist involvement *"As receptionists there's no way we would ask questions, if they [patients] did ask questions, we'd say it's best to discuss it with a nurse"* [70]	Receptionist involvement *"Ideally young people should just be able to go to the reception, pick up a kit, go to the loo, do it and then drop it back in at reception"* [67]
					Practice nurse involvement *"The question is should nurses be used that way? They could but it comes back down to who pays and if it is Medicare that pays then I am not so sure that Medicare is very comfortable with nurses initiating that and doctors signing off especially when the doctor is not really involved in the care of that patient"* [59]	Practice nurse involvement *"Look I think young people feel a lot more comfortable speaking to a nurse…I think they feel a lot more at ease"* [59]
**Psychological Capability**	Lack of patient education, knowledge, and awareness *"I think it's just general knowledge they don't really understand how bad it is do they"* [41]	Increase knowledge, education, and awareness *"… if you could almost sort of subtly slip it into… a sit-com or a popular TV show… cos it's something they can relate to"* [49]	Lack of provider knowledge and awareness *"I don't think I hardly ever do screening for chlamydia … it just doesn't occur to me. I mean if there are symptoms I would, but I am sure I should do more screening"* [64]	Increasing knowledge, awareness, and education *"The support raised my awareness and it gave me different ways of approaching young people…I think it does one good to have one's awareness raised"* [70]	Lack of testing guidance *"If we were looking at a screening policy it would have to be PCT policy. There would have to be defined budgets available and there would be a knock-on effect in terms of timing. I would have to know exactly what the resource implications were"* [64]	Prompts and reminders *"Automated reminders on computer. Some of them do actually and some don't. It'd be lovely if everybody had exactly the same system then we flag automatically 'this person is under 25', or 'it's 6 months since they had a chlamydia screen' or 'test them again now', you know, that sort of reminder"* [67]
	Forgetfulness *"Maybe forgot to get round to it, maybe, you know, sometimes you can have the best intention"* [41]		Forgetfulness *"Well I suppose we could have done [developed a prompt] but there's only three (practices) who have ours (computer system) they know (how) to deal with all those (other) sorts of computers but wouldn't have known what to do (with ours)"* [70]		Lack of knowledge, education, and general awareness *"I was under the impression that if you took a [chlamydia] swab you disturbed the cells for the [cervical] smear and conversely if you did a smear you weren't as likely to pick up chlamydia on the swab"* [65]	Testing guidelines *"Surely there's an optimum amount of testing to get a certain number of positive results… we should be looking at a clear-cut policy of who we should be testing - rather than risking upsetting patients that could happen if you did things in a more general and opportunistic way"* [64]
**Reflective Motivation**	Beliefs regarding perceived risk *"I would have had unprotected sex with men before but I just assumed I was fine. I presumed it was perfect, I was grand. This kind of immortality vibe, you're grand, you're healthy, everything is fine"* [37]	Beliefs that testing is responsible, mature, and healthy *"If you accept screening you're looking after yourself and respecting your body. I think it's been instilled in me from a young age that if you respect your body, other people will respect your body too"* [38]	Assumptions and perceptions of patients *"And it's very, very rare, as nurses we would see a male under the age of twenty-five, [an] asthma check it's the only thing that I can think of that they come to see us, or for removal of sutures"* [66]	Increase confidence *"That would come, I think, with the education. And the knowledge and awareness to have the confidence to approach and talk to people about it"* [59]	Targets set too high *"No there is no credible (target)… because first of all (they Public Health England) decide that a group of people in an area has got a certain type of infection, (and) promiscuity etc. and you say the average of the whole country is like that. (But) here*	

					it's totally different from the practice in town, how can you (set) the same target for me and B practice (in town) you cannot do that" [70]	
			Beliefs about consequences of offering *"A lot of the girls feel that you're saying you are sexually active because you're filling in this form"* [69]		Testing policy: new patients' health checks *"Some of them don't want that sort of information on their records because they might have problems and end up having hepatitis or HIV tests done at the same time. The concern is, they may [be] worried about the life insurance*" [64]	
**Automatic Motivation**	Embarrassment and shame *"I'd feel embarrassed cos then it won't be a secret. If my parents were exposed to it as well, I would be more ashamed, then I wouldn't be able to look at their face and talk to them face to face as I used"* [40]	Asymptomatic infection worries *"If you did have chlamydia there is not always noticeable symptoms and it is like a silent disease and it can make you infertile without you knowing"* [45]	Difficult to discuss *"I do find it more difficult to speak about sexual matters particularly if they come about something completely unrelated. If they come about contraception or something else it's easier. I think if they come for an ear infection, I wouldn't dream to bring it up"* [66]		Testing policy: based on behaviour *Interviewer: "How should the staff ask you to do the (chlamydia) tests?" Patient: "Ummm, I suppose not in a way that it sounds like you've been sleeping around, like*" [48]	Reward and incentive programmes *"I really don't think monthly rewards or [financial] incentives is a good idea at all. I don't agree with that at all"* [67] *"It would be helpful for us to have some incentive to carry on doing it. It's taking us away from other areas of work"* [70]
	Fear *"… cause it's, like, you know, a scary word, and especially young girls it would probably just...I know it would scare the crap out of me"* [43]					
						Feedback on efforts *"I think to keep the motivation going its essential to have feedback on how well we're doing, which we haven't had"* [70]
**Physical Opportunity**	Time constraints *"I don't think they [doctors and nurses] give you enough time to talk about anything, I feel quite rushed"* [41]	PCP offering testing *"If someone offers [it to] me, it kind of makes it easier"* [41]	Time constraints *"There is really no barrier other than a time barrier… Our own time constraints are fairly big…we never have time to scratch ourselves so from that point of view that is a definite constraint. As far as it is us fitting it into our days which could be a problem, there would be a way around it."*[59]	Mode of testing *"I think the whole procedure of taking swabs is a bit of a turn-off really"* [64]	Time constraints *"Well this time pressure makes it very difficult if someone comes in for just a normal sort of 10-minute appointment slot and you want their smear - and then counselling for chlamydia… and then they say how does it affect me and how could I get it, and you know it's 25 minutes down the line so it just makes it very difficult to put all the perfection into practice"* [64]	Promotional materials *"A poster that can be seen by everyone will be helpful, and perhaps, the invitation cards as well, so the teenagers are a bit prepared for the question. And the poster is also a reminder for the staff"*[75]
	Receptionist involvement *"I just think, with sexual health, at the end of the day, receptionists are there to book appointments, aren't they? Not to ask if you want a sexual disease test"* [44]	Mode of testing *"I've got good aim, it's still better than bloody, (the participant motions self swabbing) I ain't putting that in there!"* [40]		Receptionist involvement *"We just left [packs] on the desk at the reception and there was quite a lot taken really, we were quite surprised. We left them on the desk in a big basket with a poster about it all"* [70]	Reception area *"I think the receptionists … once again I think it depends on the area of the surgery, or like the layout maybe, because I don't think the new medical centre … you've got from, this is the couch and there's about a*	Testing policy: Inclusion in other consultations *"We see plenty of sports injuries... I tend to ask those people because its imprinted on my brain somewhere, they're at that age"* [66]

					metre, two metres before the reception desk, and I think it all depends on the layout of the room" [48]	
					Written invitations<br>*"One centre sent letters out to the under 25s, he didn't have a good response"* [67]	System to record offers<br>*"If we put on offered, but declined chlamydia screening that would be a good thing coz it would be recorded that some people have said no"* [66]
					Service cost to patient<br>*"I mean you could go to your GP and maybe get something done quicker but I went to the clinic where it was free because I couldn't afford to pay whatever to your GP like 50 or 60 euro to see him on top of whatever it costs to get those tests done"* [37]	Simplified laboratory systems<br>*"I think one of the things is addressing the pathology side of things. Like at this stage most doctors would want to be involved if there is any discussion on chlamydia. They wouldn't feel comfortable just handing that straight over to the nurses. At this stage I think the doctors would be, from what I have seen, reluctant to let us have that sort of freedom"* [59]
					Absence of systems to record test offers<br>"*We don't record if we offered it, so we haven't got a marker on our computer for that at the moment"* [66]	Support for partner notification<br>*"if we diagnose it and have to refer them for contact tracing, they're more likely to get lost"* [69]
					Lack of support for partner notification<br>*"Well I just think from a contact tracing point of view you may think they are in a stable relationship and they may not be wanting to tell you about any of the sexual encounters they may have had and they may be more willing to talk to a third party and I would guess that the GUM clinic have ways of approaching these things"* [64]	
**Social Opportunity**	Stigma<br>*"You know if you change partners then it's like I'm being really bold. It's the whole slut factor again you know. Six months later you have another new partner and you go in for another screening and then it's again the whole what are they going to think of me"* [37]	Normalisation<br>*"I would feel offended if I was singled out for testing. That seems ridiculous but I think I would honestly. It would be important to say that everyone's being tested. It would make it more normal, to say everyone's doing it. It's a bit more acceptable"* [38]	Practice social norms<br>*"I think mine's (practice) a bit ad hoc actually the screening programme... I know I definitely don't offer screens for women who are coming in for contraception... I've only offered to one young mum that I can remember"* [66]	Normalisation<br>*"I think just normalizing it, I mean, people have this stigma put onto sexual health. But, the fact of the matter is... for the most part, everyone is sexually active"*[67]	Testing policy: women only<br>*"It's just that you're insinuating something about this person. You're almost criticizing them, saying that they're a certain type of person"* [38]	Normalisation<br>*"I think the thing is it's to offer it as a general thing, as much as testing for glucose and cholesterol and demystify it a bit, for it not to be seen as some dirty word and for it to be seen as a general testing as other things are"* [66]
			Provider-patient relationship<br>*"I think it gets a bit difficult because in a country town ... one of our nurses*	Consultation social context<br>*"I think women are easier to talk to about things like that, especially*	Cultural norms<br>*"I'm [the nurse] the only one that has actually been involved in*	Testing policy: blanket testing<br>*"Yeah, I mean, if they just offered it to everyone at that age then you know*

			had four children here and they went to the local high school so their children know a lot of people in town and I think it would be a bit awkward if you had to ring up someone and said, "Look you know…" And medico-legally I mean confidentiality I always sort of worry about" [58]	*younger women, and especially you've got them in for things like smears and stuff, you know, and sometimes when they come in for things like that they tend to open up a bit more about other things"* [61]	*screening. I've got the impression when the new contract came in that the doctors weren't bothered whether I did it or not… I find as a nurse it can be quite demoralising to have an expertise that you can't use. But because of the way the surgery is [run] the uptake has been quite low"* [66]	*that you haven't been singled out, you know, it's not an embarrassing thing to … you can't change your age sort of thing [laughs], it's not because of who you are or what they think, and sort of thing"* [48]

Note: COM-B = capability, opportunity, motivation, behaviour
